# Identification of an ATP-controlled allosteric switch that controls actin filament nucleation by Arp2/3 complex

**DOI:** 10.1038/ncomms12226

**Published:** 2016-07-15

**Authors:** Max Rodnick-Smith, Su-Ling Liu, Connor J. Balzer, Qing Luan, Brad J. Nolen

**Affiliations:** 1 Institute of Molecular Biology and Department of Chemistry and Biochemistry, University of Oregon, Eugene, Oregon 97403, USA; 2 Institute of Molecular Biology, University of Oregon, Eugene, Oregon 97403, USA

## Abstract

Nucleation of branched actin filaments by Arp2/3 complex is tightly regulated to control actin assembly in cells. Arp2/3 complex activation involves conformational changes brought about by ATP, Nucleation Promoting Factor (NPF) proteins, actin filaments and NPF-recruited actin monomers. To understand how these factors promote activation, we must first understand how the complex is held inactive in their absence. Here we demonstrate that the Arp3 C-terminal tail is a structural switch that prevents Arp2/3 complex from adopting an active conformation. The interaction between the tail and a hydrophobic groove in Arp3 blocks movement of Arp2 and Arp3 into an activated filament-like (short pitch) conformation. Our data indicate ATP binding destabilizes this interaction via an allosteric link between the Arp3 nucleotide cleft and the hydrophobic groove, thereby promoting the short-pitch conformation. Our results help explain how Arp2/3 complex is locked in an inactive state without activators and how autoinhibition is relieved.

## Abstract (web summary)

Arp2/3 complex nucleates branched actin filaments, and is inactive in the absense of activators. Here the authors present a model of Arp2/3 autoinhibition, whereby the Arp3 C-terminal tail acts as a structural switch that blocks movement of Arp2 and Arp3 into an activated filament-like conformation.

## Graphical abstract

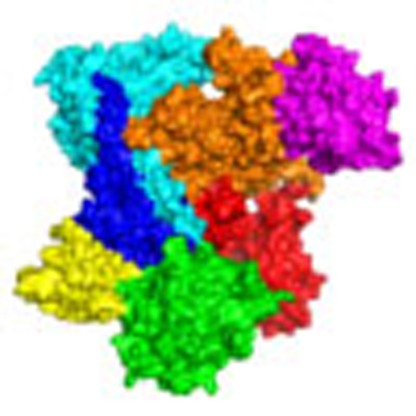

Arp2/3 (Actin-related protein 2/3) complex is a seven-subunit protein assembly that plays a key role in regulating the actin cytoskeleton. By nucleating branched actin filaments in response to cellular signals, it controls the assembly of dendritic actin networks required for a broad range of cellular processes, including endocytosis, protrusion of invadopodia and lamellipodia, and positioning of the meiotic spindles^1^,^2^,^3^. In the absence of activating factors, Arp2/3 complex is held in an inactive or low activity state. Activation typically requires interactions with several activating factors, including preformed actin filaments, actin monomers, ATP and a class of proteins called nucleation promoting factors (NPFs)^4^,^5^,^6^,^7^,^8^,^9^,^10^. The nucleation activity of Arp2/3 complex must be tightly regulated by these factors for cells to properly control the dynamics and architectures of actin networks^11^,^12^,^13^. Despite the importance of ensuring that the complex does not nucleate filaments in the absence of activators, the molecular basis by which the complex is held in an autoinhibited state is not understood.

Several high-resolution structures of autoinhibited/inactive Arp2/3 complex are available^14^,^15^,^16^,^17^. In this conformation, the two actin-related subunits in the complex, Arp2 and Arp3, align roughly end-to-end in a conformation referred to as ‘splayed' (Fig. 1). Activation is thought to require a substantial structural change in which Arp2 moves ∼25 Å into position side-by-side with Arp3, creating an Arp2–Arp3 ‘short pitch' heterodimer that mimics two consecutive actin monomers within a filament. The short-pitch conformation is hypothesized to provide the nucleus to template a new filament^14^. While this hypothesis has never been directly tested, several lines of evidence indicate that activating factors stimulate movement of Arp2 and Arp3 into the short-pitch conformation, suggesting this conformational change may be a key activation step^7^,^18^,^19^,^20^,^21^,^22^. Therefore, controlling the conformational switch from the splayed to the short-pitch state could be a critical aspect of Arp2/3 complex regulation. However, despite the availability of high-resolution structures of the inactive complex and several mutational studies, the structural features of the complex that stabilize the splayed/autoinhibited state in the absence of activators have yet to be clearly identified. Activating mutations on four Arp2/3 complex subunits (Arp2, Arp3, ARPC2 and ARPC4) have been identified either in screens or in targeted mutational analysis, but the mechanism by which these mutations influence the conformation of the complex is unknown^7^,^13^,^20^,^23^,^24^,^25^,^26^. Identification and structural dissection of autoinhibitory features would not only reveal how nucleation is prevented in the absence of activating factors, but would provide a framework for understanding how activators stimulate nucleation.

Multiple inputs (NPFs, actin monomers, actin filaments and ATP) are required for activation of the complex. Mounting evidence indicates that each of these inputs plays an important role in regulating the dynamics and architectures of Arp2/3-nucleated actin networks *in vivo*^4^,^10^,^27^,^28^,^29^. For instance, NPFs such as the WASP family proteins link Arp2/3 complex to cellular signalling pathways, while the requirement for preformed filaments ensures that the complex creates exclusively branched actin filaments^4^,^10^. Likewise, bound ATP provides a built-in timer that controls branch lifetimes, since hydrolysis of ATP occurs on the complex following nucleation and stimulates debranching^28^,^30^,^31^. Like WASP, ATP binding causes conformational changes in the complex, but how these changes are linked to activation is not known. In X-ray crystal structures, ATP binding causes the nucleotide cleft of Arp3 to close, moving subdomain 4 of Arp3 closer to subdomain 2 (refs 15, 16). ATP binding increases FRET in Arp2/3 complex tagged with fluorescent proteins, providing additional evidence for ATP-induced structural changes^21^. A precise understanding of these changes and how they are linked to activation is critical, both to determine how the ATP-binding requirement is structurally encoded into the branching nucleation mechanism and to uncover the molecular basis for regulating branched actin network dynamics.

Here we identify interactions at the Arp2–Arp3 splayed interface as key autoinhibitory features of the Arp2/3 complex. We show that destabilizing this interface stimulates population of the short-pitch conformation and activates Arp2/3 complex nucleation activity. We identify the Arp3 C-terminal tail as a switchable molecular element that controls the activity of the complex *in vitro* and *in vivo* by controlling the stability of the Arp2–Arp3 splayed interface. Specifically, our data indicate that engagement of the Arp3 C-terminal tail with the Arp3-barbed end groove helps hold the complex in the inactive splayed conformation, while release of the tail destabilizes the splayed interface to stimulate the short-pitch conformation. ATP binding stimulates the short-pitch conformation, likely through an allosteric connection between the nucleotide binding cleft (NBC) and the barbed end groove that triggers release of the Arp3 C terminus. Therefore, this work reveals a molecular mechanism by which ATP contributes to activation and provides a structural framework to understand how activators of Arp2/3 complex can relieve autoinhibition to activate branching nucleation.

## Results

### The Arp3 C terminus holds the complex in the inactive state

**Figure 2:**
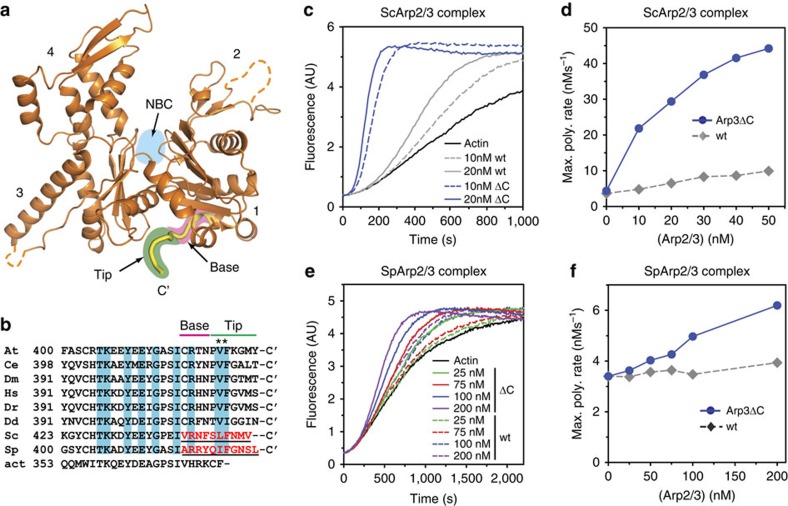
The Arp3 C-terminal tail is a conserved structural feature required for autoinhibition of Arp2/3 complex. (**a**) Ribbon diagram of Arp3 from crystal structure of *Bos taurus* Arp2/3 complex (1K8K) showing the position of the Arp3 C terminus (yellow)^14^. Subdomains 1–4 are numbered and the ATP-binding site (NBC) in Arp3 is indicated with cyan circle. The base and tip of the Arp3 C terminus are highlighted in pink and green, respectively. (**b**) Sequence alignment of the C terminus of Arp3 from diverse species. At, *Arabidopsis thalania*; Ce, *Caenorhabditis elegans*; Dm, *Drosophila melanogaster*; Hs, *Homo sapiens*; Dr, *Danio rerio*; Dd, *Dictyostelium discoideum*; Sc, *Saccharomyces cerevisiae*; Sp, *Schizosaccharomyces pombe*; act, rabbit skeletal muscle actin. Residues underlined and in red are deleted in *arp3*Δ*C* mutants. Cyan boxed residues are conserved in Arp3 sequences. Asterisks mark conserved hydrophobic residues that pin the C-terminal tail into the barbed end groove in some crystal structures. (**c**) Time course of polymerization of 3 μM 15 % pyrene-labelled actin showing constitutive activity of Arp3ΔC mutant compared with wild-type *S. cerevisae* (Sc)Arp2/3 complexes. (**d**) Maximum polymerization rate versus concentration of wild-type or Arp3ΔC *S. cerevisiae* complex for conditions described in **c**. (**e**) Time courses of pyrene actin polymerization as measured in **b**, but with *S. pombe* (Sp)Arp2/3 complexes. (**f**) Maximum polymerization rate versus concentration of wild-type or *arp3*Δ*C S. pombe* complexes for conditions described in **e**.

Both Arp2 and Arp3 contain several conserved inserts/extensions relative to actin, suggesting that in addition to mimicking actin during nucleation, the actin-related subunits play additional structural roles in the function of the complex^24^,^32^. Consistent with this hypothesis, we recently discovered two insertions within the Arp3 subunit that harbour complex-specific functions, one in controlling interactions with actin filaments and another in stabilizing attachment of a non-actin-related subunit to the complex^24^. These observations led us to wonder if other insertions/extensions could be involved in the response of the complex to its regulators. Our analysis led to one promising candidate, a conserved extension in Arp3 termed the C-terminal tail, which wedges into a cleft between subdomains 1 and 3 called the barbed end groove (Fig. 2a). The surface of the barbed end groove is largely hydrophobic, and two conserved non-polar residues within the Arp3 C terminus pin it into the groove in some structures (Fig. 2b)^14^,^16^. Molecular dynamics simulations suggested the engagement of the C-terminal tail with the barbed end groove could allosterically influence the conformation of the Arp3 nucleotide-binding cleft (NBC)^33^, so we reasoned that the tail could be important for controlling the conformation and activity of the complex. To test this, we created a budding yeast strain expressing an *arp3* mutant (*arp3*Δ*C*) lacking 10 residues from its C terminus as its sole copy of Arp3. We purified Arp3ΔC complex from budding yeast and tested its activity in a pyrene actin polymerization assay (Fig. 2c,d, Supplementary Fig. 1). The C-terminal truncation potently increased the NPF-independent activity of Arp2/3 complex. In reactions containing 50 nM Arp2/3 complex the maximum polymerization rate increased ∼20-fold on deletion of the Arp3 C terminus. These data demonstrate that the Arp3 C terminus plays a critical role in keeping the complex inactive in the absence of activators. To determine whether the autoinhibitory function of the Arp3 C terminus is conserved, we truncated the homologous residues from the C terminus of *Schizosaccharomyces pombe* Arp3. As observed with budding yeast Arp3, deletion of the C terminus of *S. pombe* Arp3 increased the NPF-independent activity of the complex (Fig. 2e,f). In the absence of nucleation promoting factors, 200 nM *S. pombe* Arp3ΔC increased the maximum polymerization rate almost two-fold over actin alone in a pyrene actin polymerization assay. Under the same conditions, wild-type Arp2/3 complex showed only a ∼16% increase in the maximum polymerization rate over reactions with actin alone. These data demonstrate that the C terminus of Arp3 has a conserved role in autoinhibiting Arp2/3 complex.

### The Arp3 C terminus influences endocytic actin assembly

**Figure 3:**
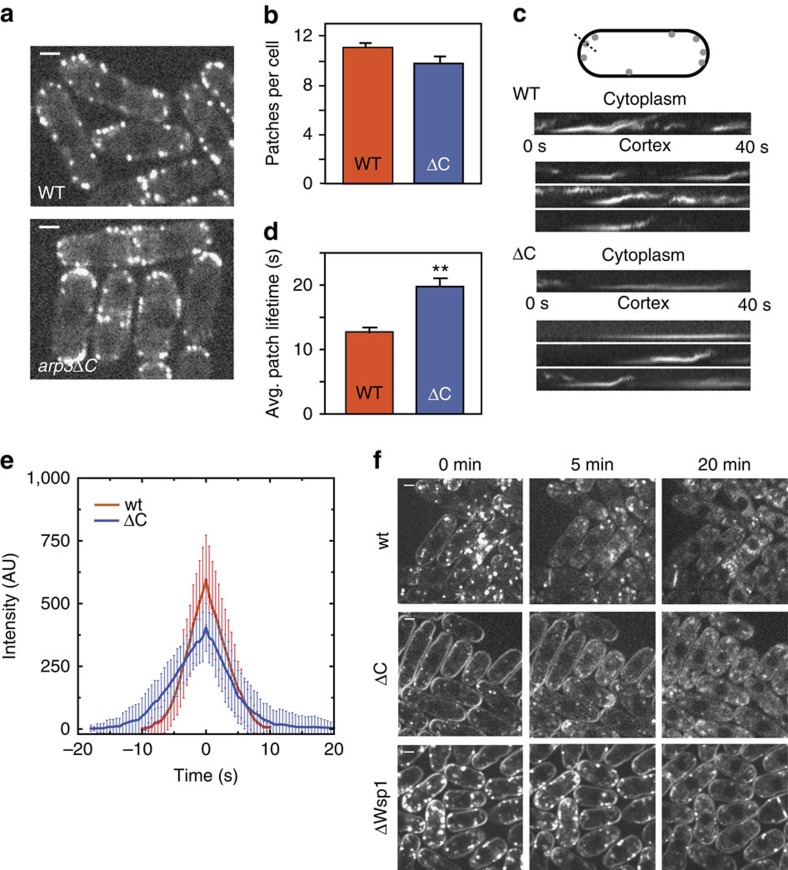
Deletion of the Arp3 C-terminal tail causes defects in endocytic actin patches and in the uptake of FM4-64. (**a**) Spinning disk confocal fluorescence micrographs of wild type (WT) and mutant *S. pombe* cells expressing Fim1-GFP. Images are taken thorough the middle of the cells. Scale bar, 2 μm. (**b**) Average number of patches per cell (*n*≥50 cells). Error bars show s.e. (**c**) Kymographs of four actin patches for wild type and *arp3*Δ*C* strain. (**d**) Average patch lifetime for wild type and *arp3*Δ*C* strains. Error bars show s.e. (**e**) Quantification of Fim1-GFP intensity in WT and *arp3*Δ*C S. pombe* cells. Data show average intensity and s.d. from measurements of patches from wt (*n*=30 patches) and *arp3*Δ*C* (*n*=30) strains, respectively. The zero time point was taken to be the frame of maximum Fim1-GFP intensity during the lifetime of each patch. (**f**) FM4-64 endocytosis assay showing that *arp3*Δ*C* strain exhibits slowed dye uptake compared with the wild-type strain. Scale bar, 2 μm.

We next asked whether deletion of the C terminus has functional consequences *in vivo*. Specifically, we tested whether deletion of the Arp3 C-terminal tail influences *S. pombe* actin patches, cortical actin networks required for plasma membrane invagination during endocytosis^7^,^23^,^34^,^35^,^36^,^37^. To monitor actin patch dynamics, we used green fluorescent protein (GFP)-tagged Fim1, an actin crosslinking protein that binds actin filaments in the patches and shows the same accumulation and disassembly kinetics as actin^38^,^39^. Actin patches in the *arp3*Δ*C* strain were similar in quantity and overall appearance to wild-type patches (Fig. 3a,b). In wild-type cells, the Fim1-GFP signal typically accumulated at the cortex and then moved inward as it disappeared, consistent with previous studies^40^,^41^. Importantly, actin patches in the *arp3*Δ*C* strain frequently failed to internalize, with only 51 % of actin patches (*n*=114) moving off of the cortex compared with 76 % of the patches tracked in wild-type cells (*n*=113, Fig. 3c). Actin patches in the *arp3*Δ*C* strain also had longer lifetimes than wild-type patches (Fig. 3d, Supplementary Movie 1 and 2). To determine whether the increased lifetimes were caused by decreased patch assembly or disassembly rates, we measured Fim1-GFP intensity throughout the patch lifetime in both wild type and *arp3*Δ*C* mutant cells. Both assembly and disassembly were slowed, and the total intensity of Fim1-GFP was less in the mutant (Fig. 3e). This result was unexpected, since increases in Arp2/3 complex activity might be expected to accelerate actin patch assembly (see discussion). We next asked if the actin defects influenced endocytosis by pulsing cells with the lipophilic dye FM4-64. In wild-type cells, the dye was immediately internalized from the plasma membrane, and stained endosomes and other internal membranes even at the first imaging time point, similar to previous reports^24^ (Fig. 3f). In contrast, uptake was delayed in the *arp3*Δ*C* strain, with strong FM4-64 staining of the plasma membrane 5 min after the initial pulse. By comparison, the delay was more pronounced in a *wsp1*Δ strain^42^, where strong staining was visible at the plasma membrane up to 20 min after the pulse. The observed defects in both actin patch dynamics and endocytosis indicate that the C-terminal tail of Arp3 is required to properly regulate Arp2/3 complex *in vivo*.

### Arp3 C terminus blocks formation of the short-pitch dimer

**Figure 4:**
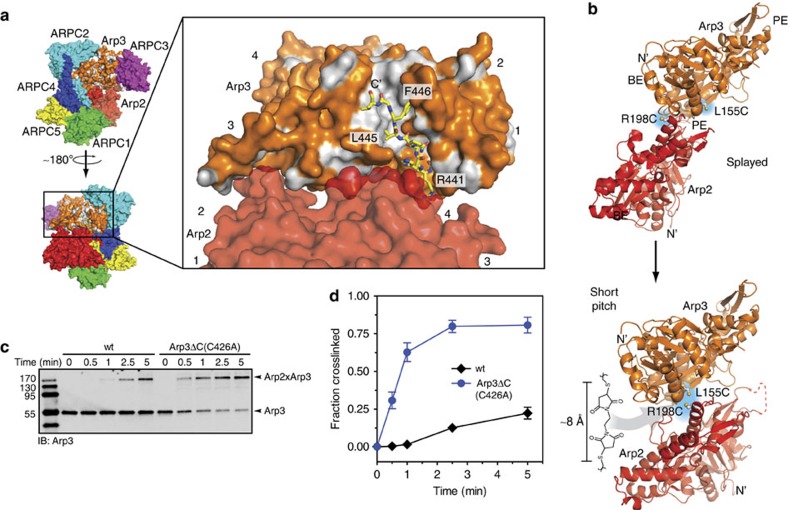
The Arp3 C terminus is required to prevent formation of the short-pitch conformation. (**a**) Surface representation of BtArp2/3 complex (4JD2) showing the barbed end of Arp3, the barbed end groove and Arp3 C-terminal tail (yellow stick representation). Residues in Arp3 are coloured according to hydrophobicity, with polar residues orange and non-polar residues grey. Residues are labelled based on the *S. cerevisiae* Arp2/3 complex sequences. (**b**) Ribbon diagrams of Arp2 and Arp3 showing the position of engineered cysteines (Arp3(L155C), Arp2(R198C)) in the splayed and short-pitch conformations and the structure of the chemical crosslinker (BMOE) used in the short-pitch crosslinking assay. Structure 4JD2 was used to make both panels. In the right panel, the Oda *et al*.^68^ actin filament structure was used to move Arp2 into the short-pitch conformation. The solvent accessible crosslinking distance between engineered cysteines is 32.5 Å in the splayed conformation, and ranges from ∼8 to 11.3 Å in different models of the short-pitch conformation (Supplementary Fig. 3)^43^. BE: barbed end; PE: pointed end. (**c**) Anti-Arp3 western blot of 1-min crosslinking reactions containing 1 μM wild type (WT) or Arp3ΔC ScArp2/3 complexes in 200 μM ATP, 1 mM MgCl_2_, 50 mM KCl, 10 mM Imidazole pH 7.0, and 1 mM EGTA. Both complexes harbour the dual-engineered cysteine residues. The Arp3ΔC complex also contains the C426A mutation to eliminate the non-short-pitch crosslinking product (Fig. 7). (**d**) Quantification of reaction described in **c**. Error bars show s.e. for three reactions.

Our data show that the Arp3 C-terminal tail plays a critical role in keeping the complex inactive in the absence of activators. We analysed the available X-ray crystal structures to determine how the tail could influence activity. The N-terminal portion of the tail, which we refer to as the ‘base' of the tail, wraps around the bottom of subdomain 1, fills the back end of the barbed end groove and directly contacts subdomain 4 of Arp2 at the splayed interface^14^,^15^,^16^. The C-terminal end of the tail, which we will refer to as the ‘tip', wedges into the front portion of the barbed end groove and is pinned into position by L445 and F446 (Fig. 4a). In some X-ray crystal structures, the tip engages the groove while in others, it is disengaged and disordered^14^,^15^,^16^. Because the base of the Arp3 C-terminal tail occupies a critical position at the Arp2–Arp3 splayed interface and the tip adopts multiple conformational states, we hypothesized that the Arp3 C terminus could be a switchable structural element that enables activating factors to influence the stability of the Arp2–Arp3 splayed interface.

To test this, we first asked whether the Arp3 C-terminal tail is required to hold the complex in the splayed conformation. We used a previously described crosslinking assay in which engineered cysteines on Arp2 and Arp3 can be crosslinked by the 8 Å crosslinker bismaleimidoethane (BMOE) only when the complex is in the short-pitch conformation^22^ (Fig. 4b). The Arp3 C-terminal deletion is not predicted to significantly influence either the solvent accessibility or the pKa of the engineered cysteine residues, ensuring that changes in short-pitch crosslinking efficiency are due to the conformational change and not the chemical reactivity of the thiol groups (Supplementary Table 1). Deletion of the Arp3 C-terminal tail in the context of the dual cysteine *S. cerevisiae* Arp2/3 complex potently stimulated crosslinking between the engineered cysteines (Fig. 4c,d). A 30-s reaction with 25 μM BMOE, 200 μM ATP and no NPF yielded 31±6 % crosslinking with the Arp3ΔC complex, while a negligible amount of the wild-type complex was crosslinked under the same conditions. These data indicate that the Arp3 C-terminal tail autoinhibits the complex by blocking it from adopting the short-pitch conformation. We note that analysis with the program X-walk showed that the minimal crosslinking length required to bond the engineered cysteines when the complex is in the short-pitch conformation (Cβ to Cβ) is between 8.0 and 11.3 Å, very close to the maximum expected crosslinking distance of BMOE (∼8 Å from sulfur to sulfur)^43^. Therefore, the crosslinked complex is held in or very near the short-pitch conformation. For simplicity, we refer to the crosslinked complex as being in the short-pitch conformation in the sections that follow, though it is important to note that we cannot currently eliminate the possibility that Arp2 and Arp3 could deviate somewhat from a perfect filament-like arrangement and still form the engineered crosslink.

### Destabilizing the splayed interface activates Arp2/3 complex

**Figure 5:**
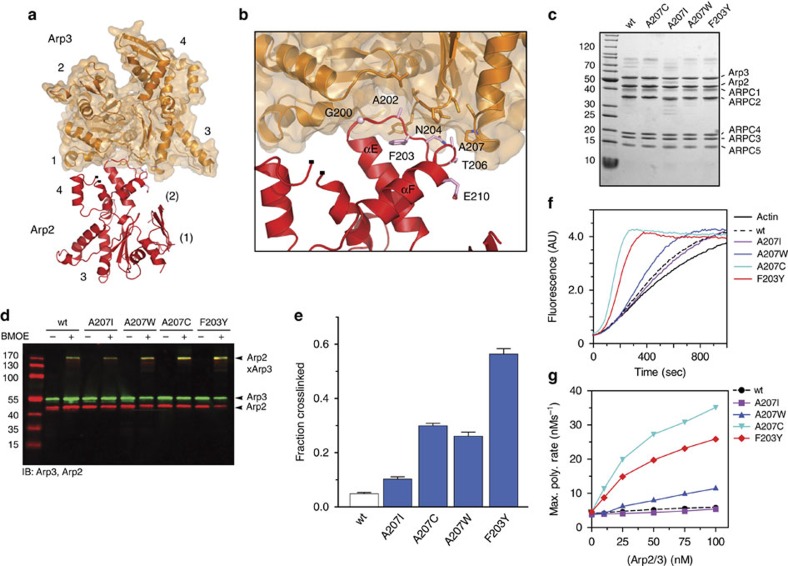
Destabilization of the splayed Arp2–Arp3 interface stimulates the short-pitch conformation and activates Arp2/3 complex. (**a**) Ribbon diagram of Arp2 and Arp3 positioned in the splayed conformation (2P9K). Subdomains are numbered, with partially or fully disordered subdomains in Arp2 in parentheses. (**b**) Close up of the Arp2–Arp3 splayed interface, with key residues from Arp2 labelled based on *S. cerevisiae* sequence. (**c**) Coomassie stained gel of purified wild type and splayed interface mutants of ScArp2/3 complex. All budding yeast complexes in this panel and in subsequent figures harbour the engineered cysteine residues: (Arp2(R198C)/Arp3(L155C)). (**d**) Anti-Arp2/anti-Arp3 western blot of 1-min crosslinking reactions with 1 μM wild type or mutant ScArp2/3 complexes in 200 μM ATP, 1 mM MgCl_2_, 50 mM KCl, 10 mM Imidazole pH 7.0 and 1 mM EGTA. (**e**) Quantification of reaction described in **d** run in triplicate. Error bars show s.e. for three reactions. (**f**) Time course of 3 μM 15% pyrene actin polymerization with 25 nM wild type or splayed interface mutant Arp2/3 complexes as indicated. No NPF is present in these assays. (**g**) Maximum polymerization rate versus concentration of wild type or mutant ScArp2/3 complexes in pyrene actin polymerization assays as described in **e**.

The base of the Arp3 C-terminal tail directly contacts Arp2 at the splayed interface, suggesting the tail prevents adoption of the short-pitch conformation by stabilizing the splayed conformation (Fig. 4a). However, some residues in the Arp3 C-terminal tail are near the Arp2–Arp3 short-pitch interface in the electron tomography model of a branch junction^44^ (Supplementary Fig. 2), so it is also possible that the C-terminal truncation influences the conformation of the complex by stabilizing short-pitch contacts between Arp2 and Arp3. To investigate these possibilities, we first sought more conservative mutations to test whether destabilization of the splayed Arp2–Arp3 interface is sufficient to stimulate the short-pitch conformation. We initially considered R441 and N442 in the base of the Arp3 C-terminal tail. These residues contribute to the splayed Arp2–Arp3 interface, but are also close to the predicted short-pitch interface (Fig. 4a, Supplementary Fig. 2). In contrast, the αE/αF loop in subdomain 4 of Arp2 contributes several residues to the Arp2–Arp3 splayed interface. None of these residues are predicted to contribute to the short-pitch interface (Fig. 5a,b, Supplementary Fig. 3)^44^. Therefore, we constructed budding yeast strains expressing Arp2 αE/αF loop mutants as their sole copy of Arp2, then purified and characterized the mutant complexes (Fig. 5c). At Arp2 position 207 we changed the wild-type alanine to bulkier amino acids to disrupt contacts of the αE/αF loop with Arp3. In addition, we mutated Arp2 F203 to Tyr. F203Y is one of two mutated residues found in an Arp2 mutant isolated in a genetic screen, *arp2-7*, that confers increased NPF-independent activity to the complex^13^,^23^,^45^,^46^. Because of its position in the Arp2 αE/αF loop, we reasoned that the F203Y mutation could also destabilize the Arp2–Arp3 splayed interface. To determine whether these mutations influence the conformation of the complex, we used the short-pitch crosslinking assay. We found that three of the splayed interface mutations increased formation of short-pitch crosslinks (Fig. 5d,e, Supplementary Table 2). In 1-min reactions, the Arp2-A207W, A207C and F203Y mutants increased crosslinking 5-, 6- and 12-fold compared with the wild-type complex, respectively. These results demonstrate that the Arp2–Arp3 splayed interface helps hold the complex in the inactive conformation. Furthermore, they show that destabilizing the splayed interface potently stimulates the short-pitch conformation. We note that the Arp2(A207I) mutant complex was prone to degradation, but when isolated showed similar behaviour in the crosslinking assay as wild-type complex, indicating not all mutations of residue 207 can stimulate the short-pitch conformation.

To determine whether the splayed interface mutations increased the NPF-independent activity of the complex, we tested the mutants in pyrene actin polymerization assays. The Arp2(A207C), Arp2(A207W) and Arp2(F203Y) mutant complexes each showed increased NPF-independent nucleation activity compared with wild-type Arp2/3 complex, consistent with their increased propensity to adopt the short-pitch conformation (Fig. 5f,g). The A207C and F203Y mutations showed the most potent activation, and at 100 nM increased the maximum polymerization rate 3.6- and 2.6-fold over the wild-type complex, respectively. The A207W mutant showed a more modest 1.8-fold increase in activity compared with the wild-type complex, and the A207I mutant was nearly identical to wild type. Altogether, our data demonstrate that destabilization of the splayed interface not only potently stimulates formation of the short-pitch conformation, but also increases the NPF-independent nucleation activity of the complex. These data support a model in which the C terminus prevents spurious activation of the complex by locking it into the splayed conformation.

### ATP binding stimulates the short-pitch conformation

**Figure 6:**
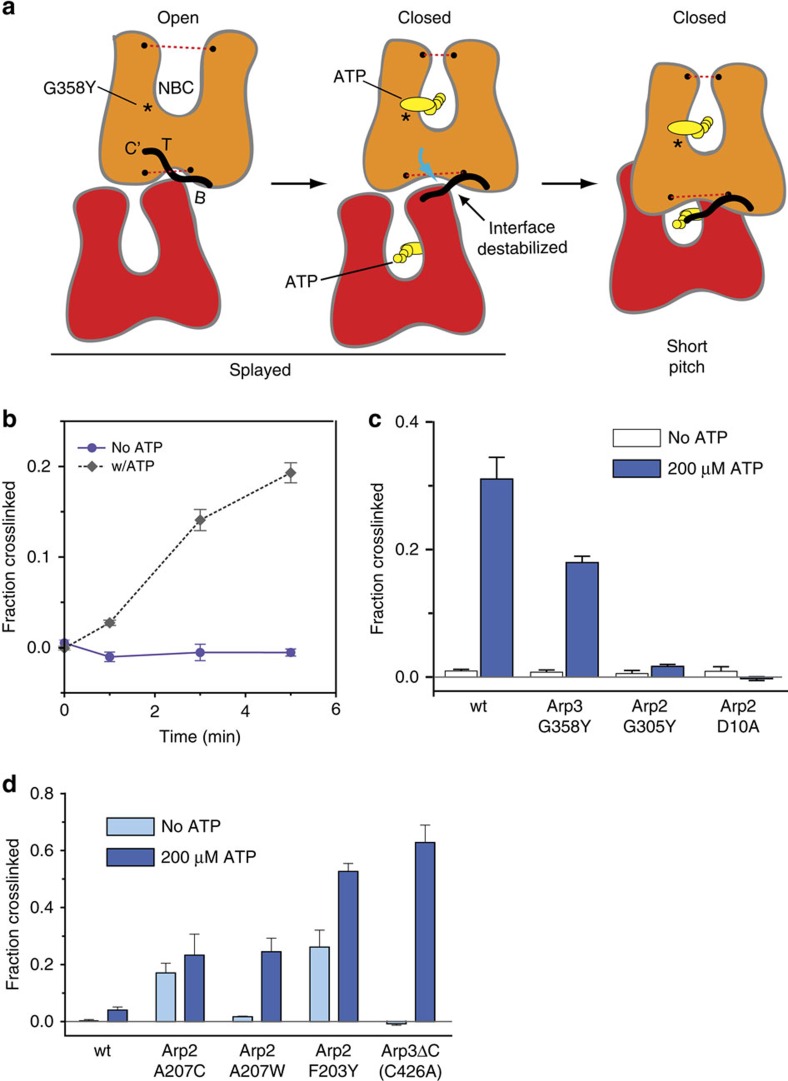
The Arp3 C terminus is an allosteric molecular switch that locks the complex in the inactive (splayed) conformation. (**a**) Cartoon depicting proposed conformational link between the ATP-binding cleft, the barbed end groove and the C terminus of Arp3. Dotted red lines indicate widths of nucleotide-binding cleft (NBC) and barbed end groove. The tip (T) and base (B) of the Arp3 C terminus are indicated. (**b**) Time course of crosslinking assays containing 1 μM wild-type ScArp2/3 complex with or without 100 μM Mg^2+^-ATP. (**c**) Quantification of 5-min short-pitch crosslinking for reactions containing 1 μM wild type or ATP-binding pocket mutation complexes with either 1 μM EDTA or 200 μM Mg^2+^-ATP. (**d**) Quantification of 1-min short-pitch crosslinking assays of wild type or splayed interface mutations with or without 200 μM Mg^2+^-ATP. Error bars in **c** and **d** show s.e. for three reactions.

The requirement for the Arp3 C-terminal tail in regulation of Arp2/3 complex *in vitro* and *in vivo*, together with evidence for structural plasticity of this feature, suggests factors that activate Arp2/3 complex could target the tail to decrease the stability of the splayed Arp2–Arp3 interface. Structural information addressing the influence of WASP family proteins on the complex is limited to low resolution EM reconstructions^7^,^19^,^20^, small angle X-ray scattering models^18^, FRET^19^,^47^, crosslinking^48^,^49^,^50^,^51^,^52^, photo-activatable label transfer-based constraints^53^, and X-ray crystal structures showing electron densities that likely represent partial segments of bound WASP. In these structures, electron density at the interface of subdomain 3 and 4 in Arp3 is consistent with three residues of the A region of N-WASP^54^,^55^. Further, tubes of electron density at the barbed ends of Arp2 and Arp3 in these structures could correspond to the predicted alpha helical segment in C^54^,^55^. The lack of complete high-resolution structures for the bound activators makes it unclear how binding of WASP could switch Arp2/3 complex from the splayed to the short-pitch state. ATP is also required for activation, and in contrast to other activators, several high-resolution structures of the complex in apo- versus ATP-bound states are available^14^,^15^,^16^. While these structures show that ATP binding closes the NBC of Arp3, Arp2 and Arp3 remain in a splayed conformation, so it is unclear how ATP could contribute to activation. However, in these structures, NBC closure is correlated with widening of the barbed end groove and release of the tip of the Arp3 C terminus^33^ (Fig. 6a). These observations led us to propose a model in which ATP helps activate the complex by stimulating closure of the Arp3 nucleotide cleft, causing release of the autoinhibitory C terminus from the barbed end groove and subsequent destabilization the splayed Arp2–Arp3 interface (Fig. 6a). As a first test of this model, we asked if binding of ATP to the complex stimulates population of the short-pitch conformation. The crosslinking assays in Figs 4 and 5 of this study were run in buffers containing ATP to match conditions of polymerization assays, so we ran a set of experiments to directly compare short-pitch crosslinking with and without ATP. These data demonstrate that ATP stimulates the short-pitch conformation in the wild-type complex. Even after five minutes of crosslinking, we did not detect any crosslinking in the absence of ATP, whereas 19±2 % of the complex crosslinked in the presence of ATP (Fig. 6b,c). ATP binds to both Arp2 and Arp3, so binding to either Arp could be responsible for stimulating the splayed to short-pitch conformational change^6^,^30^. Therefore, we tested the ability of ATP to increase short-pitch crosslinking using a previously described mutant complex defective in ATP binding to Arp3, Arp3(G358Y)^7^. Consistent with our model, the Arp3 NBC mutant Arp3(G358Y) reduced the ability of the ATP to stimulate short-pitch crosslinking, demonstrating that ATP binding to Arp3 is important for stimulating the short-pitch dimer (Fig. 6d). To probe for a role for ATP binding to Arp2, we generated two mutations in the Arp2 NBC, D10A and G302Y. Both of these mutations decrease crosslinking of ATP to the Arp2 subunit and potently decrease Arp2/3 complex activity (Supplementary Fig. 4)^7^. Unexpectedly, both mutations completely blocked ATP-induced stimulation of short-pitch conformation. Altogether, these data demonstrate that ATP binding to both Arp3 and Arp2 is important for stimulation of the short-pitch conformation, but that ATP engagement at the Arp2 NBC plays a larger role.

To further investigate the link between nucleotide binding and the stability of the Arp2–Arp3 splayed interface, we tested how activating mutations in the C terminus or in the Arp2 αE/αF loop influenced the ability of ATP to stimulate the short-pitch conformation. While the wild-type complex showed no short-pitch crosslinkling in the absence of ATP, two of the mutants, including Arp2(A207C) and Arp2(F203Y), showed significant crosslinking in the absence of ATP (Fig. 6d). Importantly, ATP-bound mutant complexes showed 8–15-fold more potent crosslinking than ATP-bound wild-type complex (Fig. 6e, Supplementary Table 3). These data indicate that the intrinsic stability of the splayed interface tunes the ability of ATP to shift the conformation toward the short-pitch state. We note that the relatively weak ATP-induced stimulation of short-pitch crosslinking in the wild-type complex is consistent with the weak NPF-independent activity of the wild-type *S. cerevisiae* complex in pyrene actin polymerization assays (Fig. 2c)^20^,^56^.

### Release of C terminus stimulates short-pitch conformation

Our data show that ATP binding to Arp3 stimulates adoption of the short-pitch dimer, even though the nucleotide-binding site is ∼20 Å from the Arp2–Arp3 splayed interface. Therefore, we wondered if structural changes initiated at the Arp3 NBC could allosterically destabilize the splayed Arp2–Arp3 interface. As mentioned above, crystal structures with ATP bound show the Arp3 cleft in a closed conformation, a state correlated with widening of the barbed end groove and disordering of the tip of the Arp3 C terminus^16^,^33^,^57^ (Fig. 6a). In contrast, crystal structures of the complex with no nucleotide bound to Arp3 tend to show an open NBC, a closed barbed end groove, and the Arp3 C terminus engaged^14^,^16^. These structural comparisons, along with molecular dynamics simulations, indicate that the NBC, the barbed end groove, and the Arp3 C terminus are allosterically linked^33^. The structural link between these regions is consistent with our measurements of etheno-ATP (ɛ-ATP) binding to the *S. pombe* complex, which specifically measures nucleotide binding to the Arp3 subunit (see Methods section). Specifically, we found that ɛ-ATP bound ∼2.7-fold more tightly to the Arp3ΔC mutant than the wild-type SpArp2/3 complex (Supplementary Fig. 5). Unexpectedly, the binding affinity of ɛ-ATP for the *S. cerevisiae* complex did not change when the Arp3 C terminus was deleted (Supplementary Fig. 5). This was surprising given that ATP binding and truncation of the Arp3 C terminus stimulate the same conformational change in ScArp2/3 complex (Fig. 4). While we cannot currently explain this result, one possibility is that ɛ-ATP engages the NBC differently than ATP so fails to form the same allosteric link to the Arp3 C terminus.

**Figure 7:**
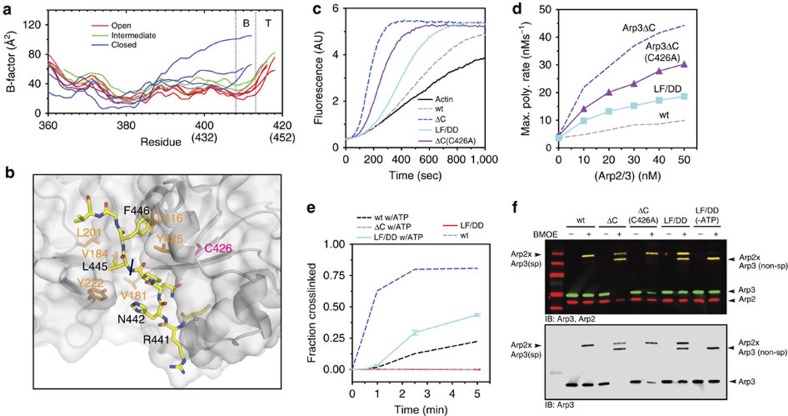
Release of the tip of the Arp3 C terminus from the barbed end groove stimulates formation of the short-pitch conformation. (**a**) Plot of average main chain B-factor versus residue number for the C-terminal residues in Arp3. Data taken from 10 different *Bos taurus* Arp2/3 complex crystal structures in different nucleotide-bound states (1K8K, 2P9L, 1U2V, 2P9N, 2P9U, 2P9P, 1TYQ, 2P9I, 2P9K and 2P9S; 14–16). Nucleotide cleft widths are classified based on Nolen and Pollard^16^. B-factors were normalized so that each chain had the same average main chain B-factor. Residues missing in the electron density are omitted from the plot. Residue numbers in parenthesis are for ScArp2/3 complex. See Supplementary Fig. 6 for more detailed B-factor analysis. (**b**) Surface representation of the barbed end groove with C terminus of Arp3 bound (yellow stick representation, black labels) based off of structure 1K8K. Orange labels indicate key hydrophobic residues in the Arp3-barbed end groove. C426, a cysteine that becomes reactive upon deletion of the Arp3 C-terminal tail, is labelled in magenta. Residue labels are based on *S. cerevisiae* sequence. Black line indicates transition between the base and tip of the C-terminal tail. (**c**) Time courses of polymerization of 3 μM 15% pyrene actin in the presence of 10 nM wild type, Arp3ΔC, Arp3ΔC(C426A) or Arp3(L445D/F446D) ScArp2/3 complexes. The C426A mutation decreased activity slightly in the Arp3ΔC complex, but was still hyperactive compared with wild type. (**d**) Maximum polymerization rate versus complex concentrations for reactions described in **c**. (**e**) Time course of short-pitch crosslinking for reactions containing 1 μM wild type, Arp3ΔC or Arp3(L445D and F446D) ScArp2/3 complexes with or without 200 μM ATP. Error bars show s.e. for at least three reactions. (**f**) Two colour (anti-Arp2/anti-Arp3) western blot of crosslinking reactions containing 1 μM wild type or mutant ScArp2/3 complexes with or without 200 μM ATP as indicated. Reactions containing wild type and Arp3(L445D/F446D) complexes with or without ATP were run for 5 min. Reactions containing Arp3ΔC or Arp3ΔC(C426A) complexes were run for 1 min.

The allosteric link between the NBC, the barbed end groove, and the Arp3 C terminus suggests that ATP binding could influence the stability of the splayed interface by controlling the engagement of the Arp3 C terminus with the barbed end groove. However, in the x-ray crystal structures, nucleotide-induced NBC closure generally results in disorder of the tip of the Arp3 C-terminal tail, while the base of the tail remains fully or partially ordered (Fig. 7a, Supplementary Fig. 6). The Arp3 C-terminal truncations we describe above remove both the tip and base of the tail (Fig. 2b). Removal of base of the tail could directly destabilize the splayed conformation because it eliminates residues that participate in the splayed Arp3–Arp2 interface (Fig. 4a). Therefore, we wondered if disruption of the tip alone could allosterically destabilize the splayed interface. To test this, we mutated to aspartate the two conserved hydrophobic residues that pin the tip into the barbed end groove (Fig. 7b). Like the complete C-terminal deletion, this mutation, Arp3(L445D/F446D), increased NPF-independent activity compared with the wild-type complex (Fig. 7c,d). In addition, the Arp3(L445D/F446D) mutation stimulated formation of the short-pitch conformation in the crosslinking assay, despite the fact that neither of the mutated residues directly contribute to the splayed interface (Figs 4a and 7e). Similar to the C-terminal deletion mutant, the Arp3(L445D/F446D) mutant required ATP for potent stimulation of the short-pitch conformation (Fig. 7e). Furthermore, like the Arp3 C-terminal truncation, the Arp3(L445D/F446D) mutant formed a secondary (non-short pitch) Arp3–Arp2 crosslinked band in the BMOE crosslinking assay (Fig. 7f). This secondary product forms due to a crosslink between an endogenous cysteine in Arp3, C426 and Arp2. C426 is at the barbed end of Arp3 adjacent to the C-terminal tail, and is reactive when the C-terminal tail is deleted, but not in the wild-type complex (Fig. 7b,d, Supplementary Fig.7). That the C426 non-short-pitch crosslink occurs in the Arp3(L445D/F446D) mutant suggests that mutation of the tip of the tail increases the solvent exposure of Arp3 C426 similarly to the C-terminal truncation, providing additional evidence that the Arp3(L445D/F446D) mutation releases the tail from the barbed end groove. Together, these data support a model in which release of the tip of the Arp3 C terminus from the barbed end groove allosterically influences the conversion from the splayed to short-pitch conformation, providing a molecular mechanism by which ATP helps relieve autoinhibition of Arp2/3 complex.

## Discussion

Our data indicate that Arp2/3 complex must be locked in the splayed conformation to prevent its spurious activation. Interactions that stabilize the splayed conformation can be described by two major sets of contacts^14^,^15^,^16^. First, Arp2 and Arp3 are held by a molecular clamp formed by ARPC1, ARPC2, ARPC4 and ARPC5 (Supplementary Fig. 8). The clamp buries ∼1,500 Å^2^ of surface area of Arp2 and 2,100 Å^2^ of Arp3. Second, Arp2 and Arp3 interact end-to-end in the splayed conformation within the clamp (Supplementary Fig. 8). The contact area of the splayed Arp2–Arp3 interface is relatively small (∼800 Å^2^), and based on analysis with the program PISA is not a relevant dimer interface in the absence of the clamp^58^. However, we show that the Arp2–Arp3 splayed interface is critical for controlling the conformation of the complex. Furthermore, our data suggest that at least one activator, ATP, targets the splayed interface to activate the complex. In addition to Arp2/3 complex activators, we note that at least one inhibitor also targets the splayed Arp2–Arp3 interface. CK-666, a small molecule Arp2/3 complex inhibitor, binds in a pocket at the splayed interface, directly stabilizing the splayed arrangement to inactivate the complex^22^. Therefore, tuning the strength of the splayed Arp2–Arp3 interface may be a general mechanism by which regulators control branching nucleation by Arp2/3 complex.

Here we present a ‘tail release' model in which the Arp3 C-terminal tail serves as a molecular switch that controls the conformation and activity of the complex. In this model, when the tip of the tail is released from the barbed end groove, it destabilizes interactions between the base of the tail and Arp2, thereby stimulating the short-pitch conformation (Fig. 6a). Existing biochemical and structural data support the tail release model, but several lines of evidence indicate that activation involves additional structural mechanisms. For instance, ATP binding increased short-pitch crosslinking in the Arp3ΔC mutant, demonstrating that deletion of the Arp3 C-terminal tail is not sufficient to completely populate the short-pitch state (Fig. 6e). In addition, while ATP binding to Arp3 contributed to stimulation of the short-pitch conformation, our data showed that ATP binding to Arp2 is actually more important, so nucleotide binding must also stimulate the short-pitch conformation through mechanisms not directly involving the Arp3 C terminus. We note that several other mutations in Arp2/3 complex are known to increase the NPF-independent nucleation activity of the complex. While the structural basis by which these mutations relieve autoinhibition is unknown, the location of the amino-acid changes suggests they may not be directly linked to the tail release mechanism^20^,^25^. Understanding how multiple structural features in the complex coordinately stimulate activation, and how Arp2/3 complex regulators target these features remain important open questions. We anticipate that the short-pitch crosslinking assay will provide an important tool in this regard.

ATP hydrolysis by Arp2/3 complex stimulates branch disassembly, so the requirement for ATP in activation creates a built-in timing mechanism to control turnover of branched actin networks^28^,^29^,^30^. Mutational analysis shows that ATP binding to both Arp2 and Arp3 is important for branching nucleation^7^,^21^, but how nucleotide binding to either Arp subunit could influence the activity of the complex was unknown. Here we show that ATP stimulates formation of the short-pitch conformation, explaining how it contributes to activation. By mutating the NBC of Arp3, we showed that ATP binding to Arp3 is important for stimulation of the short-pitch conformation, consistent with the tail release model. Previously published FRET experiments are consistent with our data, since mutations in the Arp3 NBC blocked an ATP-induced increase in FRET^21^. However, these data also showed that NBC mutations in Arp2 did not block the conformational change measured by FRET^21^, whereas the same mutations completely blocked short-pitch crosslinking in our assays here, making it unclear if the FRET assay measures the short-pitch conformational change. That ATP binding to Arp2 stimulates the short-pitch conformation is also supported by a previously described NBC mutation in Arp2, Y309A. Arp2-Y309A increases the activity of the complex both in the presence and absence of NPF^7^. In EM reconstructions of negatively stained particles, the Y309A complex favors a conformation more closely resembling the wild-type NPF-bound than free complex^7^. This suggests that it may stimulate the short-pitch conformation, and that like Arp3, the nucleotide cleft of Arp2 is allosterically linked to overall positions of Arp3 and Arp2 in the complex. Dissection of the structural basis for this allosteric link will be important for understanding how Arp2/3 complex is activated. We note that at cellular concentrations, ATP will bind rapidly to the nucleotide-free Arp subunits, so the apo-complex is likely more transient that the ADP-bound states. Therefore, determining precisely why ADP cannot activate Arp2/3 complex will also be important for understanding the nucleotide-controlled timing mechanism for branch dissociation.

Mutations that selectively uncouple the activity of Arp2/3 complex from each of its activating inputs (ATP, actin monomers, actin filaments and NPFs) will be critical to understand how Arp2/3 complex integrates activating signals to control branching nucleation *in vivo*^4^,^5^,^6^,^7^,^8^,^9^,^10^. While substantial work remains to identify new mutants and characterize existing ones, important trends have already emerged. First, despite potent *in vitro* NPF-independent activation by some mutations, the phenotype of existing activating mutations is relatively mild. For instance, deletion of the Arp3 β4/β5 insert increased activation of *S. pombe* Arp2/3 complex by WASP (Wsp1), but had no influence on endocytic actin patches^24^. Similarly, the budding yeast Arp2(Y309A) mutant showed no obvious defects in endocytic actin patch localization or organization^7^. The *arp2-7* budding yeast strain, which harbours the Arp2(F203Y) mutation we characterize here (plus one additional mutation in Arp2), showed defects in patch dynamics^13^,^23^,^45^,^46^, but like the *arp3*Δ*C* strain, the patches remained cortical and resembled wild-type actin patches. The failure of existing activating mutations to cause accumulation of ectopic actin structures suggests additional mechanisms regulate the activity of the mutant complexes *in vivo*. One possibility is that these mutations fail to uncouple the activity of the complex from the requirement to bind preformed filaments. Consistent with this hypothesis, several experiments suggest the *in vivo* activity of wild-type Arp2/3 complex is limited by the availability of suitable preformed substrate filaments^9^,^42^,^59^. Negative regulators of the complex or its activators (for example, Crn1, Syp1, Bbc1, Sla1, Lsb1) may also dampen the *in vivo* influence of the mutations, explaining the mild phenotypes^13^,^50^,^60^,^61^. A second emerging trend is that while relatively mild, the phenotypes of each of the activating mutations are distinct. These differences cannot be explained by the relative *in vitro* potency of the mutations. For instance, the *S. pombe* Arp3ΔC mutation shows modest activity *in vitro* but has the most pronounced influence on actin dynamics *in vivo*. We speculate that the precise molecular mechanism by which these mutations uncouple activation from activating inputs influences their *in vivo* effect on actin dynamics. We note that the *arp3*Δ*C* mutant is particularly intriguing in that its influence on the dynamics of the actin patches (increased Fim1 lifetime and defective internalization) is similar to what has been observed for mutations that compromise the activity of the complex rather than activate it^7^,^13^. We anticipate that further dissection of this and other key Arp2/3 complex mutants will provide important insight into the *in vivo* function of the complex.

## Methods

### Construction of fission yeast strains

A fission yeast strain harbouring the *arp3*Δ*C*(416-427) mutation was made by transforming the TP150 strain with a cassette harbouring a premature stop codon and the KanMX6 marker^62^. *FIM1* was tagged with GFP by homologous recombination strategy using a cassette generated from pFA6a-GFP-NatMX6 (ref. 63). Both integrations were confirmed by amplifying and sequencing the entire regions. See Supplementary Table 4 for all fission yeast strains used in this study.

### Construction of budding yeast strains

Splayed interface mutations in Arp2 were constructed by Quick Change PCR using as a template a previously constructed vector in which *arp2(R198C)* was subcloned into pRS317 with native promotor and terminator sequences^22^. Arp3(L445D/F446D), Arp3ΔC^440-449^ and Arp3ΔC^440-449^(C426A) expression plasmids were created by the same method, using a previously described template in which *arp3(L155C)* is flanked by native promoter and terminator sequences was cloned into pRS315. These plasmids were introduced into haploid strains with the corresponding Arp subunit knocked out and rescued on a *URA* plasmid. Haploid strains expressing Arp subunits off of the pRS plasmids were then mated, sporulated and selected through random spore analysis to generate the final yeast expression strains^22^. See Supplementary Table 5 and 6 for all budding yeast plasmids and strains used in this study.

### Protein purification and labelling

Pyrene-labelled rabbit skeletal muscle actin was purified by extracting actin monomers with G-buffer (2 mM Tris pH 8.0, 0.2 mM ATP, 0.5 mM DTT, 0.1 mM CaCl_2_, 1 mM NaN_3_) and polymerized by adding 50 mM KCl and 2 mM MgCl_2_. After 1 h, [KCl] was increased to 800 mM to promote dissociation of actin-binding proteins and then pelleted for 2 h at 32 K rpm in a F37L rotor (Thermo scientific). The actin pellet was resuspended in G-buffer and dialysed for ∼3 days against G-buffer, changing the buffer multiple times before gel filtering on a 300-ml Sephacryl S300-HR column. To label the actin with pyrene, a portion of the unlabelled actin was repolymerized in labelling buffer (25 mM Tris pH 7.5, 100 mM KCl, 2 mM MgSO_4_, 0.3 mM ATP, 3 mM NaN_3_) and labelled overnight with a 7:1 molar excess of *N*-(1-Pyrene)Iodoacetamide (10 mM stock in DMF). The pyrene-labelled actin was pelleted, and the resuspended actin pellet was dialysed and gel filtered similar to the unlabelled actin. For expression of *S. pombe* Arp2/3 complex, *S. pombe* cells were grown in 200 ml of YES medium at 30 °C. After ∼24 h, 10 ml of this culture was used to inoculate 1 l cultures of YES, which were grown at 30 °C for 12–16 h. An additional 35 g of YES was added to each 1-l culture, and cells were grown to an optical density at 600 nm of ∼6.0 before harvesting. To produce *S. cerevisiae* cell pellets for protein purification, a starter culture grown in synthetic media lacking Leu, Lys, His and Trp was added to 1-l YPD medium and allowed to grow to OD_600_ ∼7–8. An additional 50 g L^−1^ YPD powder was added and cells were harvested at OD_600_ ∼15–16. Before collecting, 2 mM EDTA and 0.5 mM PMSF was added to prevent proteolysis. Cells were pelleted in a F8B rotor at 5K rpm for 5 min. Cells were resuspended in lysis buffer (20 mM Tris pH 8.0, 100 mM NaCl, 1 mM EDTA, 1 mM DTT), and stored at −80 degrees Celsius. *S. pombe* Arp2/3 complex was purified using an ammonium sulfate cut, a GST-N-WASP-VCA affinity column, a MonoQ anion ion exchange column (GE Healthcare), and a Superdex 200 gel filtration (GE Healthcare). *S. cerevisiae* Arp2/3 complexes were purified using an ammonium sulfate cut, a GST-N-WASP-VCA affinity column and a gel filtration step. Unlike the other complexes reported here, the Arp3ΔC mutant complexes could not be eluted from a GST-N-WASP-VCA affinity column using high salt. These complexes were instead eluted with 50 mM reduced glutathione solution. The pooled fractions were then purified by monoQ and glutathione sepharose columns to remove any contaminating GST-N-WASP-VCA. Like the wild-type complexes, the Arp3ΔC complexes were gel filtered as a final purification step before flash freezing in liquid nitrogen. Anti-GST (Genescript A00865) western blots were used to demonstrate that there was no detectable GST-N-WASP-VCA in the complexes prepared with the modified purification method (Supplementary Fig. 9).

### Actin polymerization assays and kinetic data analysis

Pyrene actin polymerization assays were carried out by adding 1 μl of 100 × antifoam (100X: 0.005% Antifoam-204, Sigma) and 2 μl of 10X ME buffer (10X: 5 mM MgCl_2_, 20 mM EGTA) to 20 μl of a 5X actin stock and incubated for 2 min. Polymerization was initiated by adding 77 μl of KMEI (final concentration: 10 mM Imidazole pH 7.0, 50 mM KCl, 1 mM MgCl_2_, 1 mM EGTA, 200 μM ATP, 1 mM DTT) with and without Arp2/3 complex where indicated. Fluorescence changes were monitored by exciting the sample at 365 nm and measuring the emission at 407 nm using a Tecan Safire 2 plate reader. The maximum rate of polymer formation was calculated using the equation:


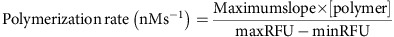


Where the concentration of polymer is equal to the equilibrium concentration (total actin minus 0.1 μM).

### Microscopy

Confocal microscopy of *S. pombe* cells was carried out at 30 °C using a Yokagawa CSU10 spinning-disc head. Cells were maintained in the exponential phase for 2 days before immobilization on a gelatin pad. Images were collected using Micomanager software and actin patches were tracked manually in Image J^64^,^65^. For patch internalization measurements, patches were tracked from multiple cells in 3 different movies, using custom Matlab particle tracking scripts^66^. Patches that moved 2 pixels (∼0.2 μm) from their origin were classified as internalized, even if the patch movement was not perpendicular to the cell cortex. To quantify the intensity of patches over time, a circle with a diameter of seven pixels was drawn in ImageJ over isolated patches and the intensity was measured for each frame. The background was subtracted by measuring the intensity of a circle of the same size in the cytoplasm of the cells. Due to the frequent patch internalization failures in the Arp3ΔC strain, patches were aligned to the maximal intensity of Fim1-GFP, which typically reflects the time at which patch movement starts to occur^40^,^67^. FM4-64 uptake assays were carried out by growing cells in YE5S to midexponential phase and incubating at room temperature with 20 μM FM4-64 for one minute. Cells were washed with fresh medium before placing on a gelatin pad and imaging.

### ɛ-ATP-binding assays

ɛ-ATP binding was measured by titration of a solution of 0.5 μM wild type or mutant fission yeast Arp2/3 complex in 10 mM Hepes pH 7.0, 50 mM KCl, 1 mM EGTA, 1 mM MgCl_2_, 1 mM DTT, 1.4 % acrylamide with ɛ-ATP and measuring the fluorescence at 420 nm (excitation=340 nm). The background fluorescence of unbound ɛ-ATP was subtracted by assuming a linear relationship between the signal and ɛ-ATP at saturating concentrations. Data were fit to the following equation:


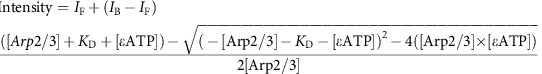


Where *I*_B_ and *I*_F_ are the intensity values of fully bound and free ɛ-ATP, respectively. We note that while both actin-related subunits in the complex bind nucleotide, previous experiments showed that mutations in the Arp2-binding pocket that potently decreased crosslinking of ATP to Arp2 had no influence on the nucleotide affinity measured by this assay, indicating ɛ-ATP measures binding specifically to the Arp3 subunit^7^.

### Short-pitch crosslinking assays

All crosslinking assays were performed with budding yeast Arp2/3 complex containing the *arp2(R198C)* and *arp3(L155C)* mutations. Reaction mixes containing Arp2/3 complex with or without additional components (for example, ATP or NPF) in KMEI were mixed with 4 μl of 125 μM BMOE for indicated times at 21 °C before adding SDS–PAGE loading buffer (1xSDS-PAGE buffer: 0.3 % SDS, 33 mM DTT, bromophenol blue) to stop the reaction. Reactions mixes were analysed by SDS–PAGE and western blotting with goat anti-Arp3 (Santa Cruz, sc-11973, 1:1,000 dilution), goat anti-Arp2 (Santa Cruz, sc-11969, 1:1,000 dilution) or mouse anti-Arp3 (Santa Cruz, sc-376625, 1:1,000 dilution) antibodies. Blots were imaged using a LICOR Odyssey Fc system.

### Structural modeling

Models used for surface area and pKa calculations were constructed by first creating homology models of ScArp2 and ScArp3 with SWISS-Model (http://swissmodel.expasy.org) using 4JD2 as a template. Arp2 was then moved into the short-pitch conformation using the Oda, *et. al*. filament model^68^. Specifically, Arp3 was superposed onto an actin subunit and Arp2 was then overlaid onto the subunit in the short-pitch position relative to Arp3. The geometry of the structure was minimized using Phenix before running the calculations^69^.

### Data availability

The data that support the findings of this study are available from the corresponding author upon request.

## Additional information

**How to cite this article:** Rodnick-Smith, M. *et al*. Identification of an ATP-controlled allosteric switch that controls actin filament nucleation by Arp2/3 complex. *Nat. Commun.* 7:12226 doi: 10.1038/ncomms12226 (2016).

## Supplementary Material

Supplementary Information Supplementary Figures 1-9, Supplementary Tables 1-7, Supplementary References

Supplementary Movie 1 Spinning disk confocal fluorescence microscopy movie of fission yeast expressing Fim1p-GFP and wild type Arp2/3 complex. Frames are taken at 0.5 sec intervals and length of entire movie is 120 seconds.

Supplementary Movie 2 Spinning disk confocal fluorescence microscopy movie of fission yeast expressing Fim1p-GFP and Arp3ΔC Arp2/3 complex. Frames are taken at 0.5 sec intervals and length of entire movie is 120 seconds

## Figures and Tables

**Figure 1:**
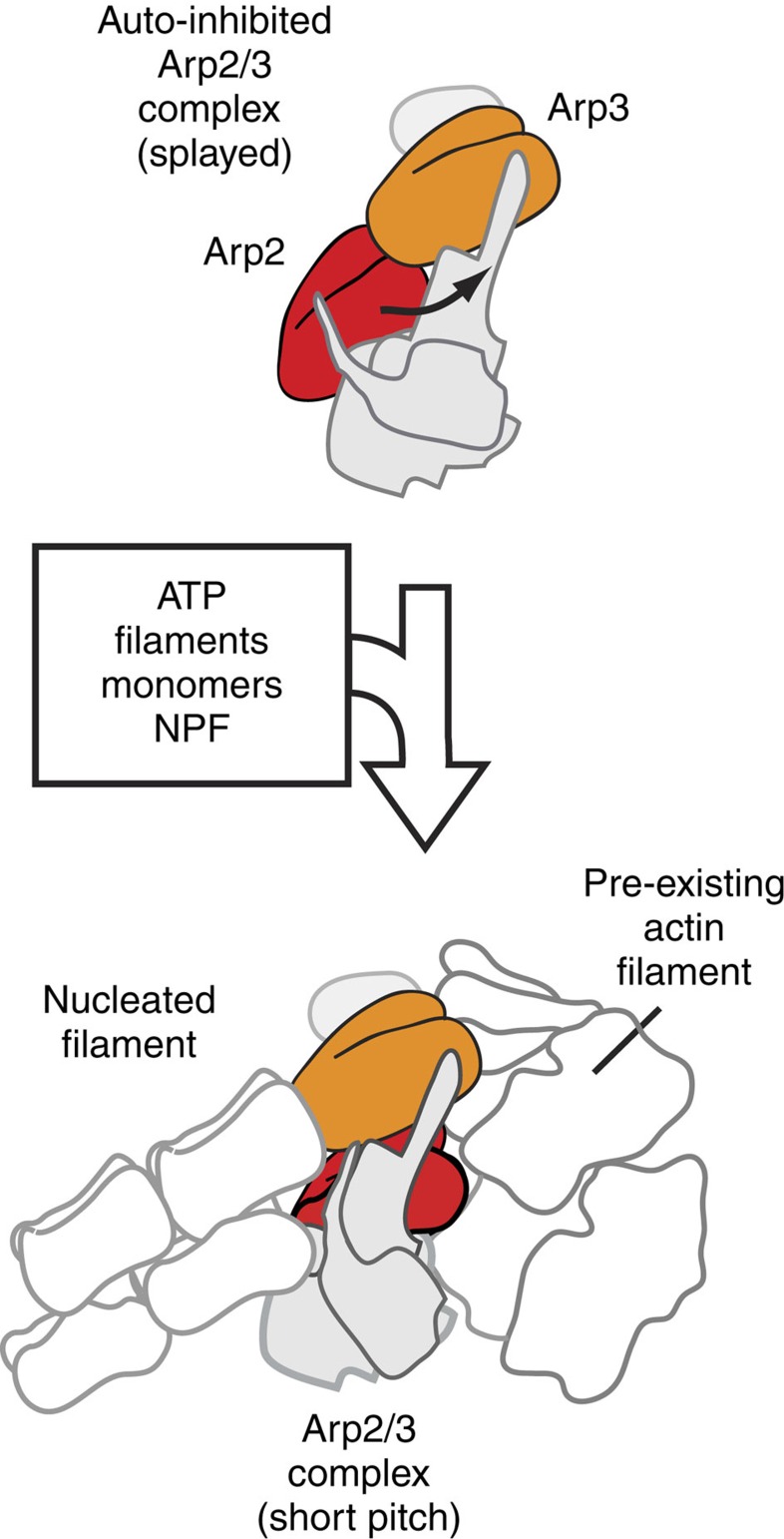
Cartoon model of Arp2/3 complex activation. Activation of Arp2/3 complex is thought to require movement of the actin-related subunits Arp2 and Arp3 from an inactive ‘splayed' conformation to an active conformation that mimics a filament-like short-pitch actin dimer. ATP, actin monomers, actin filaments and an NPF protein are typically required for activation.
