# Supplementary material for: Identification of an ATP-controlled allosteric switch that controls actin filament nucleation by Arp2/3 complex
Source: Nat Commun. 2016 Jul 15;7:12226. doi: 10.1038/ncomms12226 (PMC4947185; doi:10.1038/ncomms12226)
Supplement: Supplementary Information — Supplementary Figures 1-9, Supplementary Tables 1-7, Supplementary References [file ncomms12226-s1.pdf]

Supplementary Data

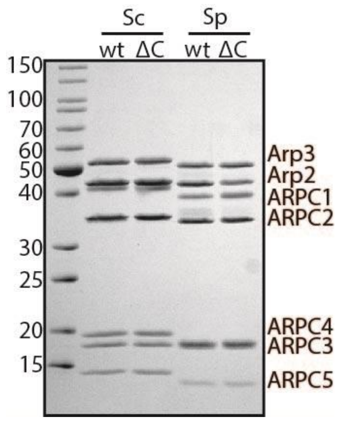

Supplementary Figure 1 | Coomassie-stained gel of purified wild type and Arp3ΔC complexes from budding and fission yeast.

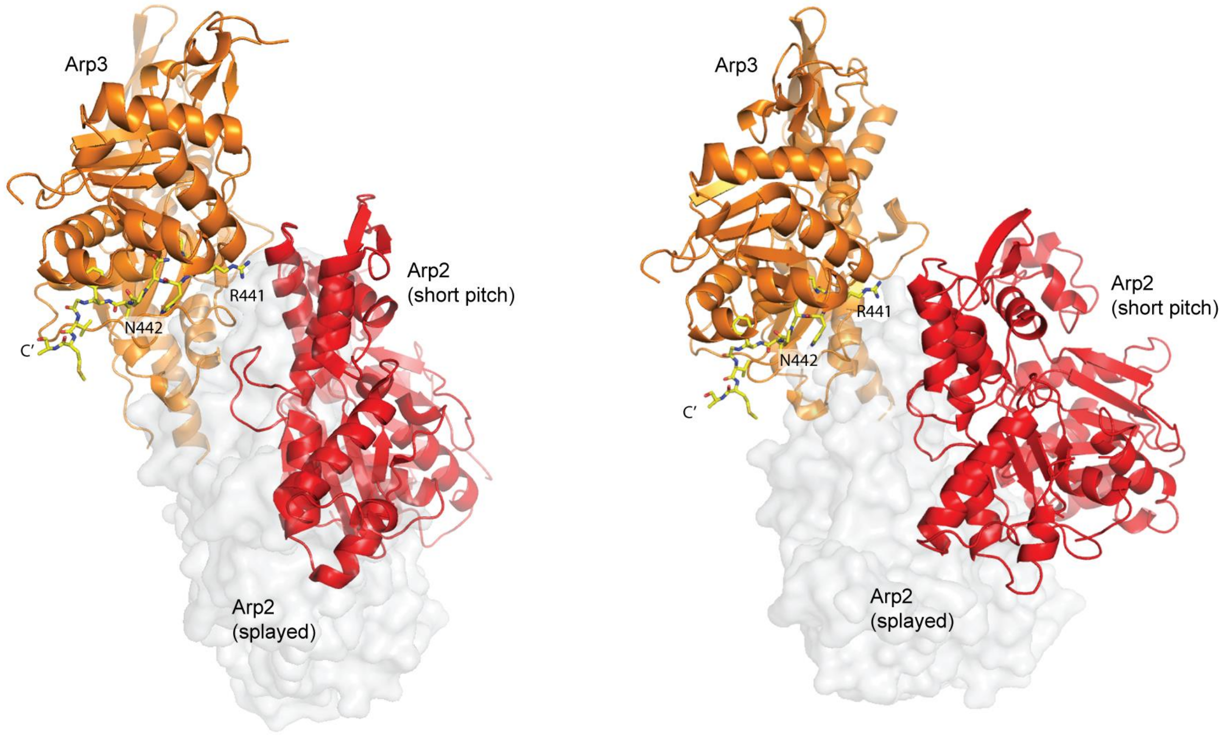

Supplementary Figure 2 | Analysis of potential contacts of Arp3 C-terminus with Arp2/3 complex in the short pitch conformation at a branch. (Left panel) Model of Arp3 and Arp3 in the short pitch and splayed conformations. The hypothetical short pitch model was created by superposing the actin filament model of Oda, et al (2ZWH) (1) onto the Arp3 subunit of 1K8K. The splayed model shows Arp2 from 4JD2 overlaid onto Arp2 from 1K8K. The Arp3 C-terminus is shown in yellow stick representation. R441 and N442 (ScArp3 residue IDs are used in this figure) are key residues from the base of the Arp3 C-terminal tail that contact Arp2 in the splayed conformation. (Right panel) Same analysis as in left panel, except that all subunits are taken from the electron tomography model of Arp2/3 complex at a branch junction (2). Arp2 is further from Arp3 in this model.

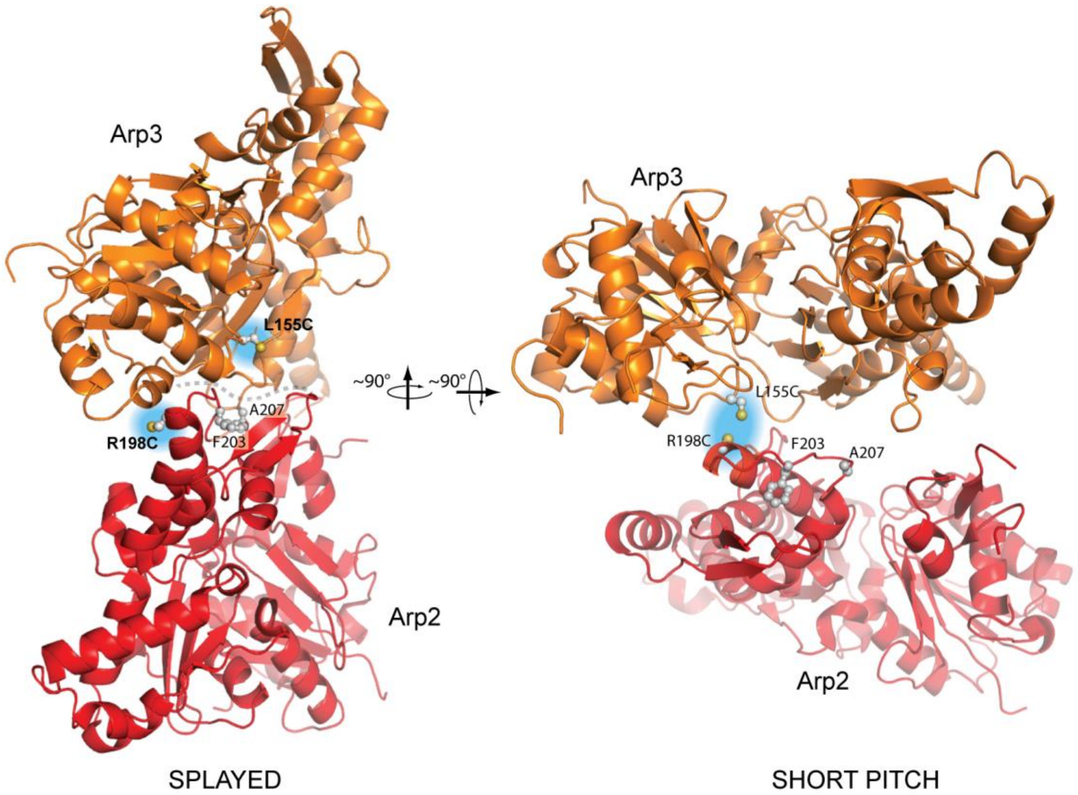

Supplementary Figure 3 | Ribbon diagram showing positions of engineered cysteine residues and splayed interface mutations in splayed or short pitch conformations. (Left panel) Arp2/3 complex in the splayed conformation from the structure of Arp2/3 complex bound to GMF (4JD2) (3). Splayed interface mutations and engineered cysteine residues are shown in ball-and-stick representation. Engineered cysteine residues (highlighted in cyan clouds) are on opposite sides of the complex in the splayed conformation and the solvent accessible crosslinking distance is 32.5 Å (Cβ-Cβ)(4). Dashed line shows the approximate location of the splayed Arp2-Arp3 interface. (Right panel) Hypothetical model of Arp2/3 complex in the short pitch conformation. Model was constructed by overlaying actin filament from Oda, et al, (1ZWH) (1) onto Arp3 from 1K8K and moving Arp2 from 4JD2 into the short pitch position. The solvent accessible crosslinking distance between the engineered cysteine residues is 8 Å in this model (Cβ-Cβ). The electron tomography structure of Arp2/3 complex at a branch junction shows similar overall positioning of Arp2 and Arp3 but using that model the solvent accessible crosslinking distance between the engineered cysteines is 11.3 Å (2)

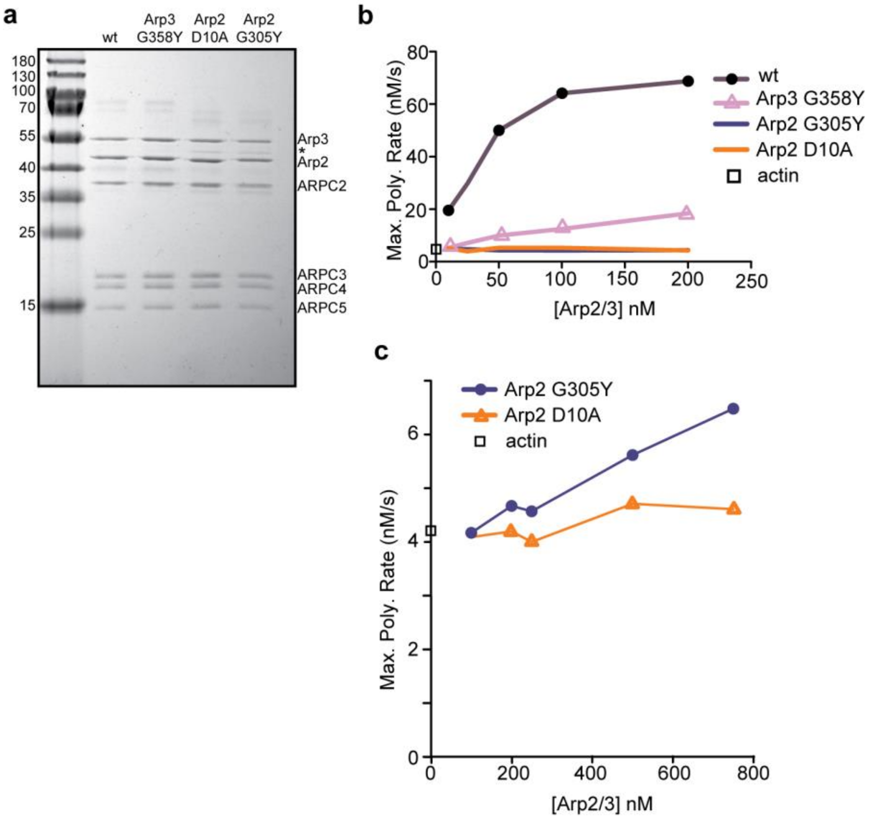

Supplementary Figure 4 | Purification and activity of Arp2 and Arp3 nucleotide binding cleft (NBC) mutant Arp2/3 complexes. (**a**) Coomassie stained gel of purified wild type and NBC *S. cerevisiae* mutant Arp2/3 complexes. Asterisks indicates possible Arp3 degradation as previously reported (5). (**b**) Maximum polymerization rates calculated from time courses of pyrene actin polymerization for reactions containing 3 μM 15% pyrene actin, 250 nM N-WASP-VCA, 500 μM ATP and the indicated concentrations of ScArp2/3 complex. Note that all complexes harbor the dual cysteine residues for short pitch crosslinking. (**c**) Maximum polymerization rates versus Arp2/3 complex concentration for reactions identical to b, except higher concentrations of Arp2/3 complex were used.

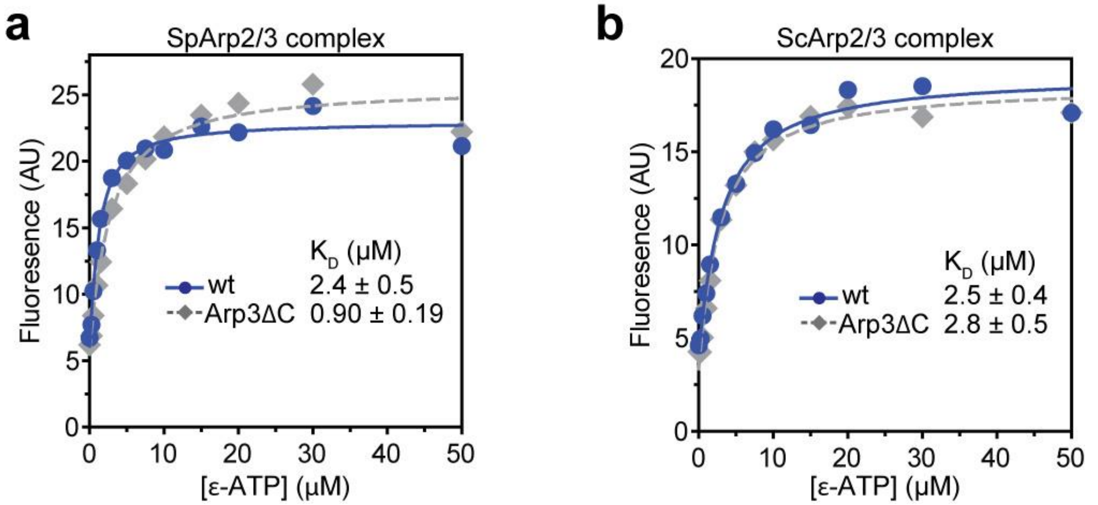

Supplementary Figure 5 | ε-ATP binding assays. Plot of background subtracted fluorescence versus ε-ATP concentration for reactions containing 0.5 μM *S. pombe* (**a**) or *S. cerevisiae* Arp2/3 complex (**b**). Data were fit assuming one binding site as described in the methods.

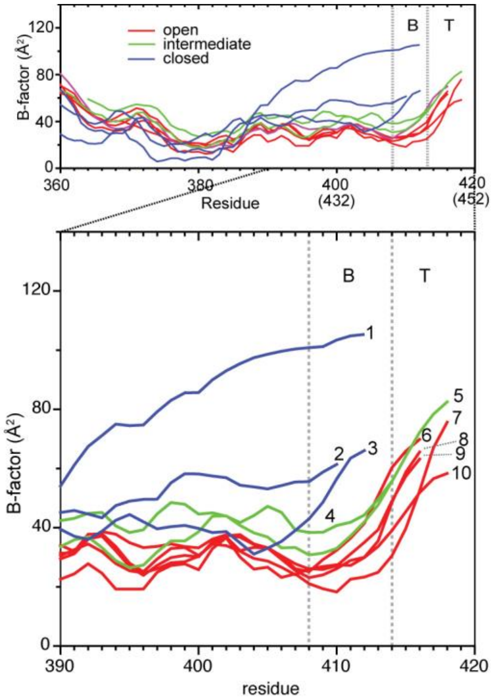

	code	ligand	G15-Cα/ D172-Cα distance(Å)	category
1	2P9I	ADP/Ca	5.5	closed
2	2P9K	ATP/Ca	5.7	closed
3	2P9S	ATP/Mg	5.6	closed
4	2P9P	ADP/Ca	6.2	intermediate
5	1TYQ	ATP/Ca	6.2	intermediate
6	2P9U	ANP/Ca	7.0	open
7	1U2V	ADP/Ca	8.0	open
8	2P9N	ADP/Ca	7.6	open
9	2P9L	APO	7.1	open
10	1K8K	APO	7.7	open

Supplementary Figure 6 | The width of the nucleotide binding cleft is correlated to the release of the C-terminal tail in Arp2/3 complex crystal structures. (related to Fig 7a in the main text) Left panels show a plot of average main chain B-factor versus residue number for the C-terminal residues in Arp3. Table on right indicates distances across the cleft, ligand bound, and classification of cleft closure. Data are taken from ten different Bos taurus (Bt) Arp2/3 complex crystal structures in different nucleotide-bound states (1K8K, 2P9L, 1U2V, 2P9N, 2P9U, 2P9P, 1TYQ, 2P9I, 2P9K, 2P9S) (6-8). Nucleotide cleft widths are classified based on Nolen and Pollard (8). B-factors were normalized so that each chain had the same average main chain B-factor. Residues missing in the electron density are omitted from the plot. Residue numbers in parenthesis are for ScArp2/3 complex. B – base of Arp3 C-terminal tail, T- tip of C-terminal tail.

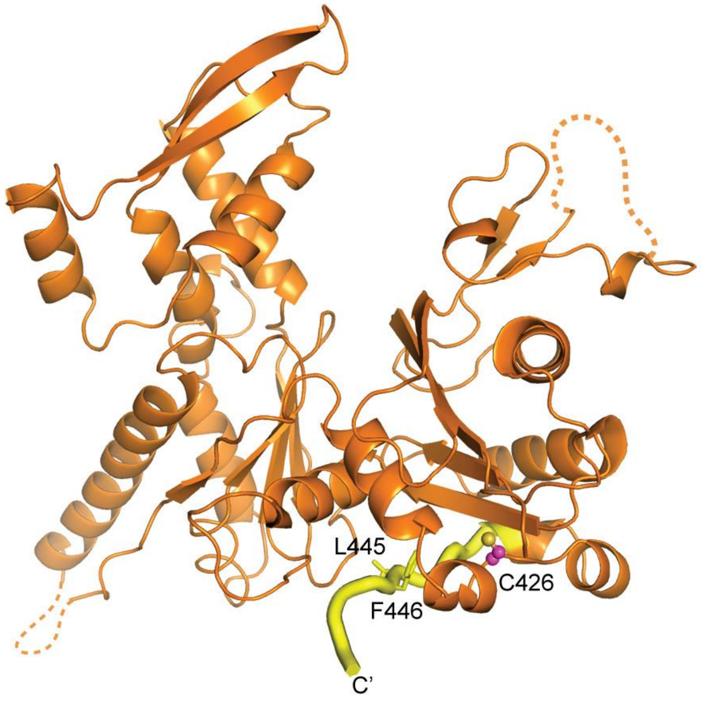

Supplementary Figure 7 | An endogenous cysteine in Arp3, C426, becomes exposed and reactive upon deletion or mutation of the Arp3 C-terminal tail. Structure of Arp3 from BtArp2/3 complex crystal structure (4JD2) showing the C-terminal tail (yellow), the two conserved hydrophobic residues in the tail (L445/F446 in *S. cerevisiae* Arp3, yellow sticks) and C426 (ball and stick).

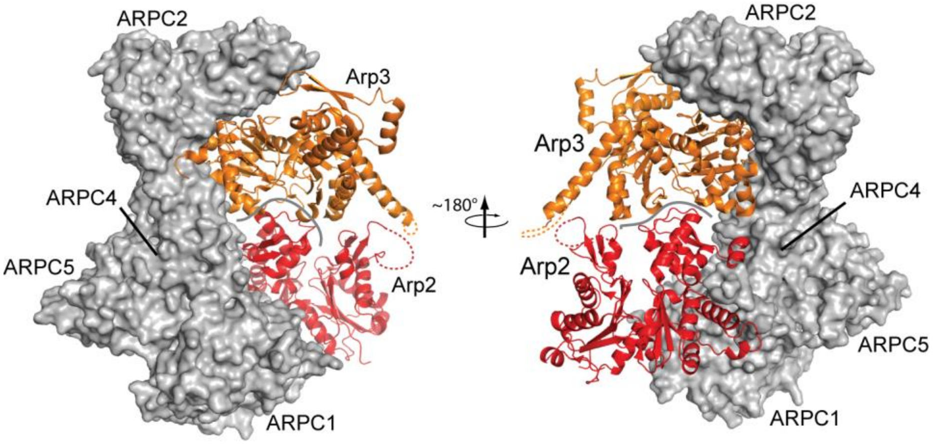

Supplementary Figure 8 | Structural visualization of contacts that hold Arp2/3 complex in the splayed conformation in the absence of activators. Structure of Arp3 and Arp3 in the short pitch conformation from the x-ray crystal structure of GMF-bound BtArp2/3 complex (4JD2) (3). ARPC1, ARPC2, ARPC4 and ARPC5 form a clamp (grey surface representation) that contacts Arp2 and Arp3 in the splayed conformation. The approximate location of the splayed interface between Arp2 and Arp3 is indicated with a solid grey line.

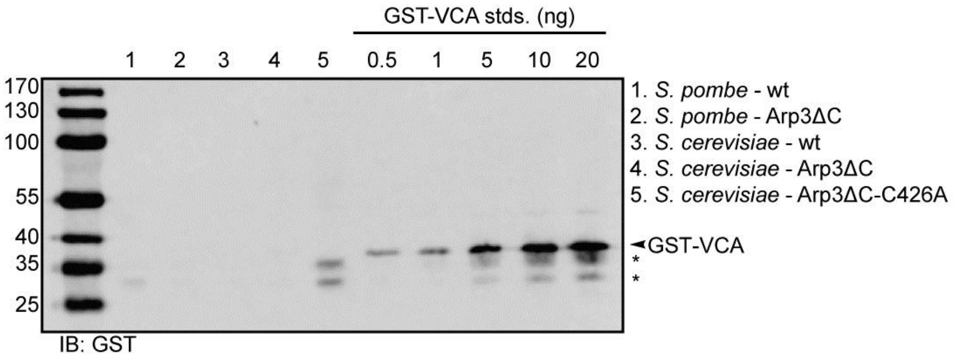

Supplementary Figure 9 | Validation of purification procedure for Arp3ΔC complexes. Anti-GST Western blot of Arp3ΔC *S. pombe* (Sp) and *S. cerevisiae* (Sc) Arp2/3 complexes verses GST-VCA standard. No GST-VCA is detectable in any of the purified complexes. Under the conditions of the loading, GST-VCA band in any of the purified samples (1-5) with the same intensity as the 0.5 ng GST-VCA standard would correspond to ~0.7 nM dimeric GST-VCA in a 1 μM solution of the purified complex. Sample 5 was not re-purified through the glutathione sepharose affinity column because no full length GST-VCA contaminant was present. We conclude that the lower molecular weight bands (asterisks) are C-terminal truncations of GST-VCA because 1.) They elute from the monoQ much earlier than full length GST-VCA, consistent with truncation of the acidic region and 2.) They bind to the glutathione sepharose affinity column.

Supplementary Tables

Supplementary Table 1 | Calculated pKa and solvent accessible surface areas for engineered cysteine residues in the context of the short pitch conformation of Arp2/3 complex.

	predicted pKa	SASA (Å)^2
**WT**		
Arp3-L155C	> 12.0	98.7
Arp2-R198C	> 12.0	53.4
**F203Y**		
Arp3-L155C	> 12.0	98.7
Arp2-R198C	> 12.0	53.4
**A207C**		
Arp3-L155C	> 12.0	98.7
Arp2-R198C	> 12.0	53.4
**A207W**		
Arp3-L155C	> 12.0	98.7
Arp2-R198C	> 12.0	53.4
**Arp3ΔC**		
Arp3-L155C	> 12.0	98.7
Arp2-R198C	> 12.0	53.4

SASA = solvent exposed surface area. pKa calculations were carried out using H++ and SASA calculations were made with POPS (9, 10)

Supplementary Table 2 | Table of p-values for Figure 5E.

	**WT**	**A207C**	**A207W**	**A207I**	**F203Y**
**WT**		p < 0.001	p < 0.001	0.049	p < 0.001
**A207C**			n.s.	p < 0.001	p < 0.001
**A207W**				p < 0.001	p < 0.001
**A207I**					p < 0.001
**F203Y**					

n.s. = non-significant. p-values calculated as two-tailed t-test comparing means of at least three experiments.

Supplementary Table 3 | Table of p-values for Figure 6C.

		WT		Arp3 (G358Y)		Arp2 (G305Y)		Arp2 (D10A)	
		No ATP	With ATP	No ATP	With ATP	No ATP	With ATP	No ATP	With ATP
**WT**	No ATP		p < 0.001	n.s.	p < 0.001	n.s.	n.s.	n.s.	n.s.
	With ATP			p < 0.001	0.02	p < 0.001	0.001	0.001	p < 0.001
**Arp3 (G358Y)**	No ATP				p < 0.001	n.s.	n.s.	n.s.	n.s.
	With ATP					p < 0.001	p < 0.001	p < 0.001	p < 0.001
**Arp2 (G305Y)**	No ATP						n.s.	n.s.	n.s.
	With ATP							n.s.	n.s.
**Arp2 (D10A)**	No ATP								n.s.
	With ATP								

Suplementary Table 4 | Table of p-values for Figure 6D.

		WT		A207C		A207W		F203Y		Arp3ΔC (C426A)	
		No ATP	With ATP	No ATP	With ATP	No ATP	With ATP	No ATP	With ATP	No ATP	With ATP
**WT**	No ATP		0.036	0.009	0.037	0.040	0.007	0.013	p < 0.001	0.024	0.004
	With ATP			0.062	0.023	n.s.	0.014	0.022	p < 0.001	n.s.	0.006
**A207C**	No ATP				n.s.	0.012	n.s.	n.s.	0.0014	0.035	n.s.
	With ATP					0.044	n.s.	n.s.	0.021	n.s.	n.s.
**A207W**	No ATP						0.009	0.015	p < 0.001	n.s.	0.004
	With ATP							n.s.	0.007	0.018	n.s.
**F203Y**	No ATP								0.016	0.028	n.s.
	With ATP									p < 0.001	0.033
**Arp3ΔC (C426A)**	No ATP										0.009
	With ATP										

n.s. = non-significant. p-values calculated as two-tailed t-test comparing means of at least three experiments.

Supplementary Table 5 | List of fission yeast strains used.

Strain	Genotype	Source
BN024	*leu1-32 sm902 arp3Δ416-427::kanMX6*	This study
BN052	*leu1-32 sm902 arp3Δ416-427::kanMX6 mEGFP-fim1::natMX6*	This study
TP150	*h^- leu1-32 sm902*	M. Yanagida
TP194	*h^- ade6-M216 leu1-32 his3-D1 ura4-D18 Δwsp1::kanMX6*	V. Sirotikin

Supplementary Table 6 | List of budding yeast expression plasmids used.

**Name**	**Gene**	**Vector**
pDW6	*ARP2*	pRS316
pDW20	*ARP3-MYC-HISx6*	pRS316
pCE04	*ARP2*	pRS305
pCE05	*ARP3*	pRS305
pBN01	*ARP2*	pRS317
pBN02	*ARP3*	pRS315
pBN03	*arp2 R198C*	pRS317
pBN09	*arp3 L155C*	pRS315
pMR01	*arp2 R198C, A207C*	pRS317
pMR02	*arp2 R198C, A207I*	pRS317
pMR03	*arp2 R198C, A207W*	pRS317
pMR04	*arp2 R198C, F203Y*	pRS317
pMR09	*arp3ΔCterm440-449, L155C*	pRS315
pMR14	*arp3ΔCterm440-449, L155C,C426A*	pRS315
pMR17	*arp3 L155C,L445D,F446D*	pRS315
pMR18	*arp2 D10A, R198C*	pRS317
pMR20	*arp2 R198C, G305Y*	pRS317
pMR22	*arp3 L155C, G358Y*	pRS315

Supplementary Table 7 | List of budding yeast strains used.

Strain	Genotype	Source
KEBY88	*MATα, ura3-52, his3-Δ200, leu2-3, lys2-801, trp1-901, suc2-Δ9, pep4-3*	T. Stevens
RLY188	*MATa, ura3-52, his3-Δ200, leu2-3, lys2-801, Δarp3::HIS3, pDW20::URA3*	R. Li
BN002	*MATa, ura3-52, his3-Δ200, leu2-3, lys2-801, trp1-901, Δarp2::TRP1 pDW6::URA3*	(11)
BN021	*MATα, ura3-52, his3-Δ200, leu2-3, lys2-801, trp1-901, Δarp2::TRP1 Δarp3::HIS3 pBN09::LEU2, pBN03::LYS2*	(11)
MR001	*MATα, ura3-52, his3-Δ200, leu2-3, lys2-801, trp1-901, Δarp3::HIS3, pDW20::URA3*	This study
MR002	*MATa, ura3-52, his3-Δ200, leu2-3, lys2-801, trp1-901, Δarp2::TRP1, pMR01::LYS2*	This study
MR003	*MATa, ura3-52, his3-Δ200, leu2-3, lys2-801, trp1-901, Δarp2::TRP1, pMR02::LYS2*	This study
MR004	*MATa, ura3-52, his3-Δ200, leu2-3, lys2-801, trp1-901, Δarp2::TRP1, pMR03::LYS2*	This study
MR005	*MATa, ura3-52, his3-Δ200, leu2-3, lys2-801, trp1-901, Δarp2::TRP1, pMR04::LYS2*	This study
MR010	*MATα, ura3-52, his3-Δ200, leu2-3, lys2-801, trp1-901, Δarp3::HIS3, pBN09::LEU2*	This study
MR012	*MATα, ura3-52, his3-Δ200, leu2-3, lys2-801, trp1-901, Δarp3::HIS3, pMR09::LEU2*	This study
MR016	*MATa, ura3-52, his3-Δ200, leu2-3, lys2-801, trp1-901, Δarp2::TRP1, pBN03::LYS2*	This study
MR017	*MATα, ura3-52, his3-Δ200, leu2-3, lys2-801, trp1-901, Δarp2::TRP1, Δarp3::HIS3, pBN09::LEU2, pMR01::LYS2*	This study
MR018	*MATα, ura3-52, his3-Δ200, leu2-3, lys2-801, trp1-901, Δarp2::TRP1, Δarp3::HIS3, pBN09::LEU2, pMR02::LYS2*	This study
MR019	*MATα, ura3-52, his3-Δ200, leu2-3, lys2-801, trp1-901, Δarp2::TRP1, Δarp3::HIS3, pBN09::LEU2 pMR03::LYS2*	This study
MR020	*MATα, ura3-52, his3-Δ200, leu2-3, lys2-801, trp1-901, Δarp2::TRP1, Δarp3::HIS3, pBN09::LEU2 pMR04::LYS2*	This study
MR022	*MATα, ura3-52, his3-Δ200, leu2-3, lys2-801, trp1-901, Δarp2::TRP1, Δarp3::HIS3, pBN03::LYS2 pMR09::LEU2*	This study
MR030	*MATα, ura3-52, his3-Δ200, leu2-3, lys2-801, trp1-901, Δarp3::HIS3, pMR14::LEU2*	This study
MR032	*MATa, ura3-52, his3-Δ200, leu2-3, lys2-801, trp1-901, Δarp2::TRP1, Δarp3::HIS3, pBN03::LYS2, pMR14::LEU2*	This study
MR034	*MATa, ura3-52, his3-Δ200, leu2-3, lys2-801, trp1-901, Δarp2::TRP1, pMR18::LYS2*	This study
MR035	*MATa, ura3-52, his3-Δ200, leu2-3, lys2-801, trp1-901, Δarp2::TRP1, pMR20::LYS2*	This study
MR037	*MATα, ura3-52, his3-Δ200, leu2-3, lys2-801, trp1-901, Δarp3::HIS3, pMR22::LEU2*	This study
MR040	*MATα, ura3-52, his3-Δ200, leu2-3, lys2-801, trp1-901, Δarp2::TRP1, Δarp3::HIS3, pBN003::LYS2, pMR17::LEU2*	This study
MR041	*MATα, ura3-52, his3-Δ200, leu2-3, lys2-801, trp1-901, Δarp2::TRP1, Δarp3::HIS3, pBN003::LYS2, pMR18::LEU2*	This study
MR042	*MATa, ura3-52, his3-Δ200, leu2-3, lys2-801, trp1-901, Δarp2::TRP1, Δarp3::HIS3, pBN003::LYS2, pMR20::LEU2*	This study
MR044	*MATa, ura3-52, his3-Δ200, leu2-3, lys2-801, trp1-901, Δarp2::TRP1, Δarp3::HIS3, pBN003::LYS2, pMR22::LEU2*	This study

Supplementary References

1. Oda, T., Iwasa, M., Aihara, T., Maeda, Y. & Narita, A. The nature of the globular- to fibrous-actin transition. *Nature* **457**, 441-445, doi:10.1038/nature07685 (2009).
2. Rouiller, I. *et al.* The structural basis of actin filament branching by the Arp2/3 complex. *J Cell Biol* **180**, 887-895 (2008).
3. Luan, Q. & Nolen, B. J. Structural basis for regulation of Arp2/3 complex by GMF. *Nat Struct Mol Biol* **20**, 1062-1068, doi:10.1038/nsmb.2628 (2013).
4. Kahraman, A., Malmstrom, L. & Aebersold, R. Xwalk: computing and visualizing distances in cross-linking experiments. *Bioinformatics* **27**, 2163-2164, doi:10.1093/bioinformatics/btr348 (2011).

5. Martin, A. C. *et al.* Effects of Arp2 and Arp3 nucleotide-binding pocket mutations on Arp2/3 complex function. *J Cell Biol* **168**, 315-328 (2005).
6. Robinson, R. C. *et al.* Crystal structure of Arp2/3 complex. *Science* **294**, 1679-1684 (2001).
7. Nolen, B. J., Littlefield, R. S. & Pollard, T. D. Crystal structures of actin-related protein 2/3 complex with bound ATP or ADP. *Proc Natl Acad Sci U S A* **101**, 15627-15632 (2004).
8. Nolen, B. J. & Pollard, T. D. Insights into the influence of nucleotides on actin family proteins from seven structures of Arp2/3 complex. *Mol Cell* **26**, 449-457 (2007).
9. Anandakrishnan R, Aguilar B, Onufriev AV. H++ 3.0: automating pK prediction and the preparation of biomolecular structures for atomistic molecular modeling and simulations. *Nucleic Acids Res*. **40,** W537-41 (2012)
10. Cavallo L, Kleinjung J, Fraternali F. POPS: A fast algorithm for solvent accessible surface areas at atomic and residue level. *Nucleic Acids Res.* **31,** 3364-3366 (2003)
11. Hetrick B, Han MS, Helgeson LA, Nolen BJ. Small molecules CK-666 and CK-869 inhibit actin-related protein 2/3 complex by blocking an activating conformational change. *Chem Biol.* **20,** 701-712. (2013)
